# Regulation and safety measures for nanotechnology-based agri-products

**DOI:** 10.3389/fgeed.2023.1200987

**Published:** 2023-06-21

**Authors:** Ritika Kumari, Kalpana Suman, Swagata Karmakar, Vandana Mishra, Sameer Gunjan Lakra, Gunjan Kumar Saurav, Binod Kumar Mahto

**Affiliations:** ^1^ University Department of Botany, Ranchi University, Ranchi, Jharkhand, India; ^2^ Bioresources and Environmental Biotechnology Laboratory, Department of Environmental Studies, University of Delhi, Delhi, India; ^3^ Department of Environmental Studies, Ram Lal Anand College, University of Delhi, Delhi, India; ^4^ Department of Botany, J. N. College, Ranchi, Jharkhand, India; ^5^ Department of Zoology, Rajiv Gandhi University, Doimukh, Arunachal Pradesh, India; ^6^ Gut Biology Laboratory, Department of Zoology, University of Delhi, Delhi, India

**Keywords:** nanotechnology, regulation, safety measures, agri-product, nanocarrier, peptidecarrier

## Abstract

There is a wide range of application for nanotechnology in agriculture, including fertilizers, aquaculture, irrigation, water filtration, animal feed, animal vaccines, food processing, and packaging. In recent decades, nanotechnology emerged as a prospective and promising approach for the advancement of Agri-sector such as pest/disease prevention, fertilizers, agrochemicals, biofertilizers, bio-stimulants, post-harvest storage, pheromones-, and nutrient-delivery, and genetic manipulation in plants for crop improvement by using nanomaterial as a carrier system. Exponential increase in global population has enhanced food demand, so to fulfil the demand markets already included nano-based product likewise nano-encapsulated nutrients/agrochemicals, antimicrobial and packaging of food. For the approval of nano-based product, applicants for a marketing approval must show that such novel items can be used safely without endangering the consumer and environment. Several nations throughout the world have been actively looking at whether their regulatory frameworks are suitable for handling nanotechnologies. As a result, many techniques to regulate nano-based products in agriculture, feed, and food have been used. Here, we have contextualized different regulatory measures of several countries for nano-based products in agriculture, from feed to food, including guidance and legislation for safety assessment worldwide.

## Introduction

The security of food, nutrition, and energy has come under intense strain due to the climate problem, population growth, a scarcity of arable land, diminishing crop yields, and growing crop use as raw materials for industry (Rosenzweig et al., 2020). According to United Nations 2019, the demand for food will increase as the world population increases by 34% by 2050 (www.un.org). The developed crops with enhance phenotypes through conventional plant breeding methods (classical and mutational) will not be enough to meet the immediate availability of food and fodder globally. However, these methods were unable to introduce features that are not currently present in many plant species (Arya et al., 2020). Despite of setbacks, there is time to choose modern, scientific and technical advancement to redress the agricultural insufficiencies. To meet the demand, several crops were successfully improved/enhanced against biotic and abiotic stresses through plant genetic engineering via the genetic modification or genetic alteration of plants by using advanced biotechnological technique like RNA interference (siRNA and miRNA) and genome editing (CRISPR/Cas: Clustered Regularly Interspaces Palindromic Repeats/CRISPR associated protein). Nanobiotechnology has the potential to revolutionize the field of plant genetic engineering, specifically by leveraging nanocarriers to transport biomolecules into plant cells (Arya et al., 2021a). Recently, nanotechnology emerged as an advance technique for the improvement of agricultural products and play a significant impact on the world’s economy and industries (Haris et al., 2023).

Although seeds were set for this field’s research about 50 years ago, history reveals that applications of nanotechnology to agriculture have only just begun to appear (Mukhopadhyay, 2014; Chhipa, 2019; Sashidhar et al., 2019; Pramanik et al., 2020; Usman et al., 2020). In last decade, for the sustainable agriculture, several nanotechnology-based mechanisms were developed for the improvement of crops using nanomaterials (NMs) or engineered nanomaterials (ENMs) against various biotic and abiotic stresses such as nanopesticides, nanobiofertilizers, nanobiosensor and soil decontamination (Usman et al., 2020; Sonawane et al., 2021; An et al., 2022). As a result, recent years have seen a considerable increase in interest in studies pertaining to uses of nanotechnology in agriculture and, the use of nanomaterials is necessary to enhance the fertilization process, raise yields through nutrient optimization and reduce the need for plant protection agents (Huang et al., 2015; Parisi et al., 2015; Kah et al., 2019). For the delivery of nanomaterials and engineered nanomaterials in to the plant cells, the cell wall act as a physical barrier to delivering functional biomolecules due to its size exclusion limit (5–20 nm) (Zhang H. et al., 2019; Arya et al., 2021b). Conventional biomolecule approaches in plants have significant limitations such as low transgenes efficiency, a small species spectrum for application, a small variety of cargo types, and tissue injury. Cunningham et al. (2018), suggested that the advancement in nanotechnology have made it possible to get beyond constraints in traditional methods: Nanoparticles (NPs) show promise for the passive transfer of DNA, RNA, and proteins across species by enhance genetic engineering (GE) via targeted and an efficient delivery. The constraints of designing an ideal NC with a broad host range, high cargo loading capacity, and efficiency are thus the subject of great attention, to access plant cells and the potential to move inside a plant’s system without the need for external mechanical aid (Jinek et al., 2012; Burlaka et al., 2015; Cunningham et al., 2018; Demirer et al., 2019).

Nano carrier based genetically modified crops has been successfully introduced in several plants such as rice, tobacco, rapeseed, maize, wheat, onion, cotton, cowpea, spinach and arugula (Demirer et al., 2019). These nano-based agri-products need to be addressed with different disciplines and strategies to meet or evaluate any kind of hazardous (physico-chemical parameters) or negative effects in humans, animals and environment. Various countries including the United States of America, Europe, India, China, Canada, Australia, and others have developed regulatory frameworks to address genetically engineered agricultural products using nanocarriers. These regulations focus on overseeing nano-based products in the field of plant genetic engineering on a global scale. So, in the current review discussed about different regulatory measures of several countries for NC-based products in agriculture including their guidance and legislation for safety assessment throughout the world.

### Advanced approaches for biomolecules delivery

**FIGURE 1:**
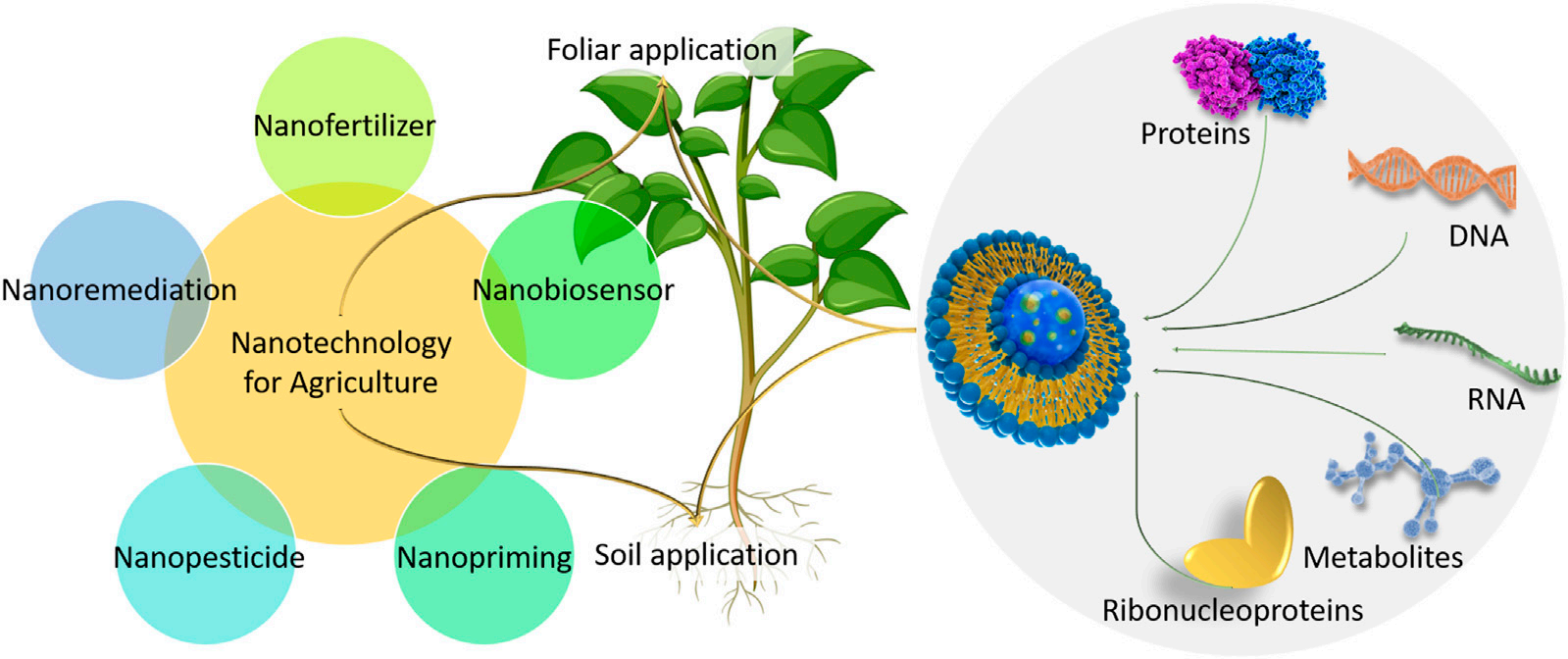
Various approaches for the delivery of biomolecules, DNA, RNA and protein using nano-/peptide-carrier. This figure was drawn by using BioRender (https://www.biorender.com).

Methods based on nanotechnology have been suggested as low-cost, simple, and reliable ways to transfer genes or other compounds into plants with great efficacy and minimal harm (Chandrasekaran et al., 2020). Biomolecules and chemicals have been successfully delivered into cells in both plant and mammalian cell systems using nanotechnology-based techniques (Ahmar et al., 2021). Genetic engineering was frequently employed in plant improvement to increase productivity and crop fitness, including yield enhancement, nutritional quality enhancement, herbicide tolerance, drought resistance, insect resistance, and viral resistance (Altman and Hasegawa, 2011; Chang et al., 2013; Wang et al., 2014; Mahakham et al., 2017; Fortuni et al., 2019; Mahto et al., 2020). According to the base material, NPs for gene delivery can be categorized as carbon nanotube-based (DNA and RNA), silicon-based (DNA and protein), metallic-based NPs (only deliver DNA as genetic cargo), or polymer-based NPs (encapsulated RNA, DNA and proteins) (Silva et al., 2010; Bates and Kostarelos, 2013; Moon et al., 2014; Kafshgari et al., 2015; Karimi et al., 2016; Zhao et al., 2017; Zhou et al., 2018; Su et al., 2019; Sashidhar et al., 2021) (Supplementary Table S1; Figure 1). The size, concentration and types of nanomaterials plays a crucial role to decide the level of toxicity. The increased surface area of the nanomaterials with decreased size shows a positive correlation with the uptake efficiency by the plants which might be responsible for the adverse effects in the system (Nel et al., 2006). It has been reported that size less than 5 nm and 20 nm can easily translocate through the pores of cell wall and plasmodesmata, respectively. This reflects that, the decreased size of nanoparticles can easily be taken up by the plant system which ultimately leads to the toxicity in the plants after accumulation (Ma et al., 2010; Rico et al., 2011; Sashidhar et al., 2021). Besides size and concentration, nanomaterials should possess few properties for its positive outcome and interaction with the plant system such as reactivity and light confinement, etc. Due to these properties, composition of nanomaterials maybe categorized as carbon nanomaterial, metal-based nanomaterial, quantum dots and nano polymers (Sun et al., 2019; Yan et al., 2021). The toxicity level of such nanomaterials can be assessed during the germination period and growth period in which the carbon nanomaterials (fullerene, carboxyfullerene, graphene oxide, etc.) and metal-based nanomaterials (Cerium, titanium, zinc oxide, etc.) had showed the desirable results in the plant system by reducing the level of toxicity as well as the negative effect. Its effects had been recorded in *Arabidopsis*, *Nicotiana*, bean, flax, etc. (Liu et al., 2010; 2013; Clement et al., 2013; Anjum et al., 2014; Cunningham et al., 2018). Another strategy to make a superior plant which is capable to cope up with the biotic as well as abiotic stress, is possible through the nano-priming technology. This technique involves the treatment of desired seeds with the nanoparticles and priming is done in nanoparticles solution. The resultant nanoprimed seeds then produces a nanoprimed plants such as *Zea mays*, *Oryza sativa* and *Triticum aestivum* with the enhanced properties at molecular, chemical and physiological level (Wang Z. et al., 2019; Afzal et al., 2021; Shah et al., 2021; Imtiaz et al., 2023). Overall, NPs should have the ability to crossing the cell wall and localise to organelles.

### Nanotechnology-based agriculture product

**FIGURE 2:**
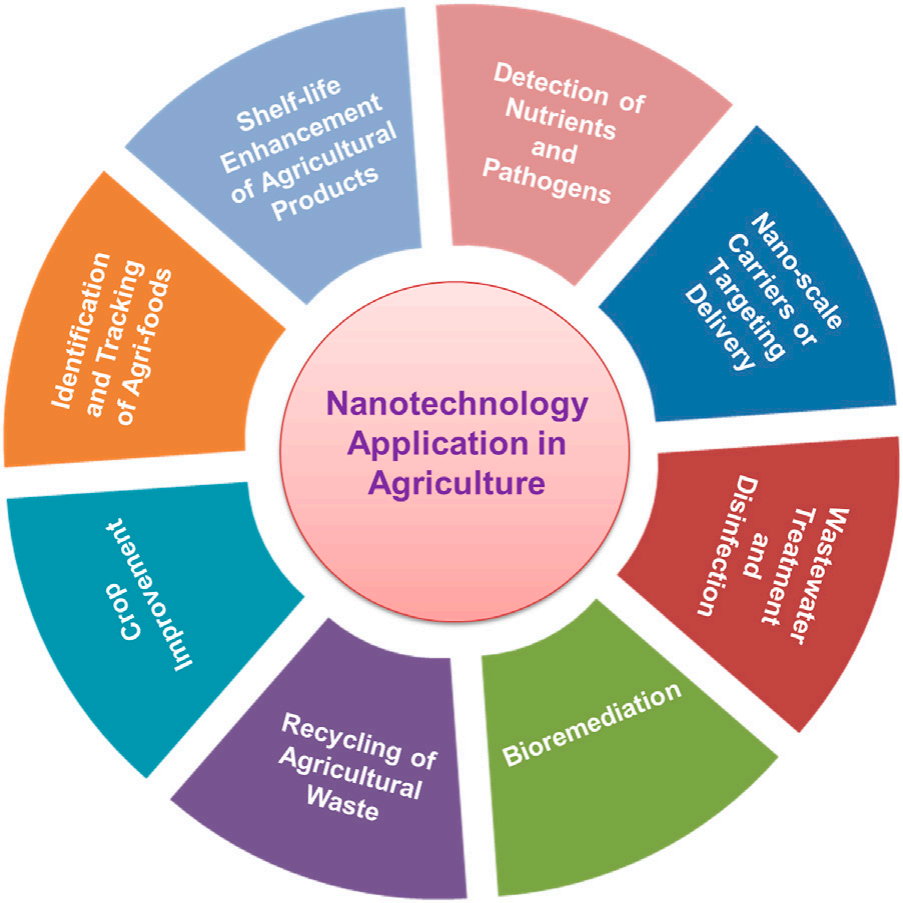
Several applications of nanotechnology in agri-food sector.

In recent years, various tools and devices created by nanotechnology, such as nanodevices and nanocapsules have been utilized to improve, diagnose and treat plant diseases, transport active ingredients to specific target areas, purify waste water, and improve plant nutrient absorption. With the increasing global population, climate change and burden on pests and diseases of agricultural crops, food security is a major concern especially in the developing nations. Nano-based agriculture products are designed and developed with aim of enhancing food security around the world. Nanotechnology is being used for synthesizing and delivering; fertilizers, pesticides, plant growth regulator, transgenic plants with disease resistance, high yield and more nutritional values (Oliveira et al., 2015; He et al., 2019) (Figure 2).

#### Nanofertilizers

Conventional fertilizers are being used indiscriminately in agriculture to keep pace with ever increasing demand of food for increasing population as conventional chemical fertilizers have lower nutrient uptake capacity and suffer high losses. Nanofertilizers (<100 nm in size) are outstanding alternative to overcome negative impact of conventional fertilizers because they reduce nutrient loss from fertilizers and application rate of fertilizers (Dimkpa and Bindraban, 2017; Babu et al., 2022). Research and development of nanofertilizers is skewed towards plant micronutirents like iron, zinc, manganese, copper, Nickel and molybdenum (Supplementary Table S2) (Dimkpa and Bindraban, 2017; Sashidhar et al., 2020; Al-Mamun et al., 2021; Arya et al., 2022; Soni et al., 2023). Nano-carbons (Biochars), carbon nano-onions and Chitosan NPs have reported to boost growth and quality of agricultural crops (Saxena et al., 2014; Tripathi et al., 2017; Khalifa and Hasaneen, 2018; Arya et al., 2022). When tomato plants were treated with Cu NPs at 250 mg L^−1^ resulted in significant increase in fruit quality and bioactive compound, whereas treatment at 500 mg L^−1^ had negative effect on bioactive compound of tomato fruits (López-Vargas et al., 2018). Joint application of silica nanoparticles (SiNPs) at 250 mg L^−1^ and 600 mg L^−1^ through soil and foliage respectively, resulted in enhancement of flowering characteristics, growth and flowering period in marigold, *Tagetes erecta* L. (Attia and Elhawat, 2021). ZnO NPs at 100–200 mg/kg improves photosynthesis of cilantro (*Coriandrum sativus*), though at 400 mg/kg affected the nutritional components of the cilantro. ZnO NPs also showed less toxicity than bilk and ionic counterparts (Pullagurala et al., 2018). CuO and ZnO NPs can traverse through many chemical and biochemical processes which could damage plant cells, affect soil biota and nitrogen fixation and even could result in critical health problem (Rajput et al., 2020). Thus, nanofertilizers have the ability to transform the agriculture, but nanoparticle related toxicity at high concentration, their accumulative effect and biosafety related comprehensive study must be done before commercialization nanofertilizers (Tripathi et al., 2017; Khalifa and Hasaneen, 2018; He et al., 2019).

#### Nanopesticides

Different types of pesticides has been used indiscriminately worldwide, owing to the growing demand of agriculture-based food product. This indiscriminate use of different pesticides, has been associated with unprecedented environmental damage due to contamination of soil, water and food, leading to harmful effect on non-target pest species and humans (Guillette et al., 2012; Oliveira et al., 2015). Thus, it is important to develop a novel technique to minimize harmful effect of pesticides, without lowering production of agricultural crops.

Nanopesticides (nanometer size range) provide a solution with its three characteristic features; to increase solubility, slow/targeted release and protection against premature degradation (Kah et al., 2013). Nanopesticides can be based on nanoemulsion, nanodispersion, solid liquid nanoparticles and nano metals (Supplementary Table S2). Silica Nanoparticles (SiO_2_-NPs, 2 g/kg of stored grain) showed 100% mortality against four stored product insects; *Rhizopertha dominica, Tribolium castaneum, Sitophilus oryzae,* and *Orizaephilus surinamenisis* (El-Naggar et al., 2020). Temperature-responsive mixed micelle (MMs–Pys–7) of pyrethrins exhibited higher larvicidal activity against *Culex pipiens pallens* at 26 °C (Zhang Y. et al., 2019). Carboxymethyl chitosan (CMCS) modified mesoporous silica nanoparticles (MSN), when loaded with azoxystrobin results in better fungicidal effect against tomato late blight *Phytophthora infestans* (Xu et al., 2018). Nanopermethrin based on nanoemulsion are more potent larvicidal than bulk permethrin (Anjali et al., 2010). Nanometal based imidacloprid has shown significantly high toxicity against *Martianus dermestoides* than aqueous formulation (Guan et al., 2008). Thus, nanopesticides are better alternative of pesticides as they more potent and required in low dosages than traditional pesticides with high toxicity against target organism and low toxicity in aquatic medium.

#### Nano-based plant growth regulators

Plant growth hormones like auxins, cytokinins, gibberellins, nitric oxide, abscisic acid, and ethylene (either synthetic or natural) are used in various ways in agriculture to improve crop production (Pereira et al., 2017). Various nanoparticles system is used for control release of plant growth hormones for most efficient and justified use as these result in sustained release of active agent as well in protecting against degradation processes (Supplementary Table S2). Nitric oxide (NO)-releasing chitosan nanoparticles (CS NPs) containing the NO donor S-nitroso-mercaptosuccinic acid (S-nitroso-MSA) allow a sustained NO release resulting in increase of NO bioactivity under salt stress in maize plants (Oliveira et al., 2016). It has been observed that when tomato plants are grown on multi-walled carbon nanotubes (CNTs) supplemented soil, they bear twice flower and fruit as compared with control plants thus CNTs acted as plant growth regulators (Khodakovskaya et al., 2013). In another study, poly (γ-glutamic acid) (γ-PGA) and chitosan (CS) polymers nanoparticles encapsulated gibberellic acid (GA3) showed increase biological activity, rate of seed germination and leaf area in *Phaseolus vulgaris* as compared to free GA3 (Pereira et al., 2017).

#### Nanosensors

Nanosensers has many beneficial aspects in agriculture such as real time monitoring of environmental conditions and stress, crop growth and diseases, pest attack and nutrient efficiency (Supplementary Table S3) (Chen and Yada, 2011; He et al., 2019). Development and advancement in the nanosensers technology had great contribution towards sustainable agriculture by real time monitoring of fertilizers and pesticides in the field, thereby reducing their excess use. Many different nanomaterials haven been used for development of nanosensors for pesticides detection; various nano composites with polymers, Carbon nanotubes (CNT), gold nanoparticles (Au NP) and quantum dots (QD). Nanosensors based on enzymes acetylcholinesterase (AChE) and/or choline oxidase (ChOx) enzyme as biological receptors for detection of organophosphorus and carbamate pesticides in smaller amount and are very sensitive in these pesticides detection (Zheng et al., 2011; Cesarino et al., 2012; Liu et al., 2012; Talarico et al., 2016; Telarico and Georgiev, 2016). Soil nutrients like nitrate have been successfully detected in lower concentration in the direct filed setting using Cysteamine modified gold nanoparticles and graphine oxide based nanosensors (Mura et al., 2015; Pan et al., 2016). Graphene-based nano-antenna integrated carbon nano-tubes sensed volatile organic compound (VOCs) emitted by plant during insect attack, hence can be used for insect attack monitoring (Afsharinejad et al., 2016). Nanosensors have been utilized for detection of water tension of soil in real time, soil pH and nutrient, prediction of nitrogen intake and detection of pathogen in soil (Bellingham, 2011; Fraceto et al., 2016).

#### Nanotechnology in transgenic plant development

For sustainable agriculture, plant genetic engineering is crucial for enhancing crop output, quality, and resilience to abiotic/biotic stressors (Shaheen and Abed, 2018). Plant genetic engineering frequently makes use of Agrobacterium, biolistic bombardment, electroporation, and poly (ethylene glycol) (PEG)-mediated genetic-transformation systems. These methods do, however, have drawbacks, such as species dependence, loss of plant tissues, ineffective transformation, and high cost (Fiaz et al., 2021). Methods of gene delivery based on nanotechnology have recently been developed for plant genetic modification (Altpeter et al., 2016; Wang J. W. et al., 2019; Zhang Y. et al., 2019). Excellent transformation efficiency, strong biocompatibility, acceptable exogenous nucleic acid protection, and the potential for plant regeneration are all demonstrated by this Nano strategy. Yet, the gene-delivery mechanism in plants that is mediated by nanomaterials is still in its infancy, and there are several obstacles to its widespread usage. The traditional methods of genetic modification applied to plants. The advancement of the development of gene delivery methods based on nanomaterials is then taken into consideration. The use of plant nanotechnology in conjunction with CRISPR-Cas-mediated genome editing is being addressed (Puchta et al., 1993; Wright et al., 2005; Christian et al., 2010). The conceptual advancements, techniques, and real-world applications of nanomaterial-mediated genetic transformation will help advance plant genetic engineering in contemporary agriculture.

Several nanoparticle-mediated transgenic delivery techniques and the plant biotechnology industry’s crowded field of existing methods. Together with a mix of the many newly created technologies, some other intriguing approaches, such the CRISPR technology, might be used in the processes of changing crops. Unfortunately, a number of significant problems still need to be fixed (Supplementary Table S4). The majority of these problems might be resolved by combining several approaches for the efficient delivery of various genomes, the design and production of contemporary hybrid NMs, and the advancement of pollen magnetofection and CRISPR techniques (Watson et al., 2018; Ahmar et al., 2021). In conclusion, while nanotechnology applications may take some time to join the area, sustained support for and knowledge of these challenges will guarantee that the field is not negatively impacted in the future.

### Safety and regulations for nanotechnology based agri-products around the world

Nanotechnology has been increasingly used in the agricultural sector for various purposes, such as enhancing crop growth, improving soil quality, and developing more efficient and targeted pesticide delivery systems (Prasad et al., 2014; Prasad et al., 2017; Hassani et al., 2020; Singh et al., 2021). However, the use of nanotechnology in agri-products raises apprehensions about the possible environmental and health risk factors related to their use (Khot et al., 2020; Mishra and Singh, 2021).

To address these concerns, regulatory bodies around the world have developed guidelines and regulations to ensure the safe use of nanotechnology in agri-products. Here are some of the key regulations and guidelines related to nanotechnology-based agri-products.

1. Regulatory Oversight: Each country has its own regulatory framework for using nanotechnology in agriculture. In the United States, Pesticides are governed by the Environmental Protection Agency (EPA), while agricultural biotechnology products are governed by the U.S. Department of Agriculture (USDA). Nanotechnology in foods and pesticides are governed by the European Chemicals Agency (ECHA) and the European Food Safety Authority (EFSA) in the European Union.
2. Risk Assessment: Regulatory bodies require risk assessment before approval of any nanotechnology-based agri-product. This includes evaluating the toxicity of the nanomaterials used, the potential for environmental release, and the impact on human health.
3. Labeling: Regulatory bodies require labeling of agri-products that contain nanomaterials. This helps consumers make informed decisions about the products they purchase and use.
4. International Standards: International standards have been developed to assure the safety and quality of nanotechnology-based agri-products. The International Organization for Standardization (ISO) has developed several standards related to nanotechnology, including ISO/TS 80004-1, which provides terminology and definitions for nanomaterials.
5. Research and Development: Regulatory bodies encourage research and development of nanotechnology-based agri-products to ensure that the products are safe for human health and the environment. Overall, the safe use of nanotechnology in agri-products requires a collaborative effort between researchers, manufacturers, regulatory bodies, and consumers. It is important to continue to monitor and assess the risks associated with nanotechnology-based agri-products to ensure their safety and effectiveness (Arya et al., 2021a). Some examples of nanomaterials that have been studied and defined by United States regulatory bodies on parameters such as safety, risk assessment, and effectiveness (Supplementary Table S5).

Different countries have established various regulations and guidelines to ensure the safe use and development of nanotechnology-based agri-products (Supplementary Table S6). Here are some examples:

***United States of America***: Nanotechnology-based agricultural products in the United States are regulated by a number of governmental organisations, including the Food and Drug Administration (FDA), the Environmental Protection Agency (EPA), and the United States Department of Agriculture (USDA). There are nanomaterials that have been approved by the US-FDA for use in food applications. The following are some examples:

1. Titanium dioxide: This is a common food additive used as a whitening and brightening agent in various food products, such as candy, chewing gum, and powdered sugar. Nanoscale forms of titanium dioxide have been approved for use in food products (Powell et al., 2016).
2. Silica: Nano-sized silica is used in some food products as an anti-caking agent, such as in powdered foods like coffee creamer (US Food and Drug Administration, 2020).
3. Zinc oxide: Nanoscale zinc oxide has been approved for use as a food colorant and as a dietary supplement (US Food and Drug Administration, 2020).
4. Iron oxide: Nanoscale iron oxide has been approved for use as a food colorant (US Food and Drug Administration, 2020).

In general, these agencies have a common goal of ensuring the safety and efficacy of nanotechnology-based agricultural products, while also ensuring that they are in compliance with applicable regulations.

Here are some key laws, safety measures, and regulations related to nanotechnology-based agri-products in the United States:

1. The Toxic Substances Control Act (TSCA): This law gives the EPA the authority to regulate the production, importation, use, and disposal of chemical substances, including nanomaterials. Nanomaterials used in agri-products fall under the TSCA, and companies are required to provide the EPA with information on the potential health and environmental effects of these materials.
2. The Federal Insecticide, Fungicide, and Rodenticide Act (FIFRA): This law regulates the registration and use of pesticides in the United States. Pesticides that contain nanomaterials must be registered with the EPA, and companies must demonstrate that the products are safe for use.
3. The Food, Drug, and Cosmetic Act (FD&C Act): The FDA regulates the use of nanotechnology in food and cosmetics. The FD&C Act requires that food and cosmetic products be safe for use and properly labelled. The FDA also requires that manufacturers of nanotechnology-based products provide information about the safety and efficacy of these products.
4. The National Organic Program (NOP): The NOP is a USDA program that regulates the use of organic labelling on agricultural products. Products that are labelled as organic must meet certain standards, including restrictions on the use of synthetic substances. The NOP does not specifically address the use of nanomaterials in organic products, but companies that produce organic products are still required to comply with all applicable regulations.
5. The Nanotechnology Research and Development Act (NRDA): This law directs federal agencies to coordinate research and development efforts related to nanotechnology. The goal is to ensure that the risks and benefits of nanotechnology are well understood and that appropriate regulations are in place.

In addition to these laws and regulations, there are several safety measures that companies can take to ensure the safety of nanotechnology-based agri-products, including.

1. Conducting rigorous safety testing: Companies should conduct thorough safety testing to identify any potential risks associated with nanomaterials used in their products.
2. Labelling: Companies should properly label their products to provide consumers with information about the ingredients used in their products. Labelling only the ingredients is a necessary but not sufficient requirement for meeting regulatory guidelines. Apart from listing the ingredients, other relevant information such as the dosage, potential exposure routes, and any associated health risks should also be provided to ensure consumer safety. For instance, in the case of nanotechnology-based agri-products, the U.S. Food and Drug Administration (FDA) recommends that companies provide additional information about the nature and properties of the nanomaterials used, such as their size, shape, and surface area, to enable risk assessment and management. Companies are also advised to evaluate the potential exposure pathways and take measures to minimize exposure to workers, consumers, and the environment (U.S. Food and Drug Administration, 2018).
3. Environmental impact assessments: Companies should conduct assessments to determine the potential environmental impact of their products.
4. Training: Companies should train employees on the proper handling and disposal of nanomaterials to reduce the risk of exposure.

Overall, the regulation of nanotechnology-based agri-products in the United States is an evolving field, and companies must stay up-to-date on the latest laws, safety measures, and regulations to ensure the safety and efficacy of their products.

***United Kingdom***: In the UK, the regulation of nanotechnology-based agri-products falls under the responsibility of several governmental agencies, including the Food Standards Agency (FSA), the Department for Environment, Food and Rural Affairs (DEFRA) and the Health and Safety Executive (HSE).

One of the primary regulations governing the safety of nanotechnology-based agri-products in the UK is the Nanotechnology Safety Guidance produced by the HSE in 2011. This guidance provides information on the safe handling and use of nanomaterials in various industrial settings, including agriculture.

The FSA is responsible for ensuring the safety and quality of food products, including those derived from nanotechnology. In 2014, the FSA published a report on the safety of nanomaterials in food, which recommended that the use of nanotechnology in food products be subject to risk assessment and evaluation. Additionally, DEFRA has issued guidance on the use of nanomaterials in agriculture, including the safe handling and disposal of nanomaterials in agricultural settings. This guidance was updated in 2018 to reflect the latest scientific knowledge on the potential risks associated with nanomaterials. Overall, the regulation of nanotechnology-based agri-products in the UK is a rapidly evolving field, with new regulations and guidance being issued on a regular basis to reflect the latest scientific understanding of the potential risks and benefits of nanotechnology.

***Europe***: Nanotechnology-based agri-products, such as pesticides, fertilizers, and animal feed additives, are subject to various regulations in Europe to ensure their safety for human health and the environment. Here are some of the key laws and regulations for nanotechnology-based agri-products in Europe, along with their reference and year (EFSA Scientific Committee, 2011; ECHA, 2012).

1. Regulation (EC) No 1107/2009 - This regulation establishes the rules for placing plant protection products on the market in the European Union (EU). It requires that all plant protection products be authorized before they can be sold or used. The regulation also sets out the data requirements for the authorization of plant protection products, including those that contain nanomaterials (Year: 2009)
2. Regulation (EC) No 396/2005 - This regulation establishes maximum residue levels (MRLs) for pesticides in or on food and feed derived from plants and animals. It also applies to pesticides containing nanomaterials (Year: 2005)
3. Regulation (EC) No 1935/2004 - The general safety standards for products and materials that come into contact with food are established by this regulation. It applies to all nanomaterials used in food contact materials, including those used in agri-products (Year: 2004)
4. Regulation (EC) No 767/2009 - This regulation establishes the rules for the authorization and marketing of feed additives in the EU. It also requires that all feed additives be safe for animals and the environment. The regulation applies to all feed additives containing nanomaterials (Year: 2009)
5. Regulation (EU) No 2019/1009 - This regulation establishes the rules for the making available on the market of CE-marked fertilizers. It also requires that all fertilizers be safe for human health and the environment. The regulation applies to all fertilizers containing nanomaterials (Year: 2019)

In addition to these regulations, there are also guidelines and recommendations from various European agencies and organizations, such as the European Chemicals Agency (ECHA) and European Food Safety Authority (EFSA), on the safety assessment of nanomaterials used in agri-products.

***Canada***: In Canada, nanotechnology-based agri-products are regulated under several laws and regulations to ensure their safety for consumers and the environment. Some of the key regulations and their corresponding references and years are.

1. Canadian Environmental Protection Act, 1999 (CEPA): This act is the primary federal legislation for regulating the environmental and human health impacts of nanotechnology-based products, including agri-products. The CEPA provides the framework for the assessment and management of nanomaterials under the New Substances Notification Regulations (CEPA, 1999; Chemicals and Polymers, 2015).
2. Food and Drugs Act (FDA): This act is Canada’s federal legislation for regulating food safety and consumer health. The FDA provides the legal framework for ensuring the safety, quality, and efficacy of food products, including those that use nanotechnology. The FDA also sets out labeling requirements for food products that use nanomaterials (FDA, 2019a; FDA, 2019b).
3. Pest Control Products Act (PCPA): This act is Canada’s primary legislation for regulating pest control products, including those that use nanotechnology. The PCPA sets out the requirements for registering and labeling pesticide products, as well as the safety and efficacy requirements for these products (PCPA, 2002).
4. Canada Agricultural Products Act (CAPA): This act regulates the marketing and inspection of agricultural products in Canada. Under this act, agri-products that use nanotechnology are subject to inspection and quality control standards to ensure their safety for consumers (CAPA, 1985).

***Australia***: The use of nanotechnology in agriculture is a rapidly growing field, and in Australia, the regulation of nanotechnology-based agri-products is overseen by several regulatory bodies. Here are some of the relevant laws, safety standards, and regulations for nanotechnology-based agri-products in Australia (Bartholomaeus, 2011).

1. Australian Pesticides and Veterinary Medicines Authority (APVMA) regulates the registration and use of agrochemical products, including those that incorporate nanotechnology. In 2014, the APVMA released a guidance document on the regulation of nanomaterials in pesticides and veterinary medicines (APVMA, 2014).
2. Food Standards Australia New Zealand (FSANZ) is responsible for regulating the safety and labelling of food products, including those that use nanotechnology. In 2015, FSANZ published a risk assessment of titanium dioxide nanoparticles in food (FSANZ, 2015).
3. Work Health and Safety (WHS) laws in Australia require that employers take reasonable steps to ensure the safety of workers who may be exposed to nanomaterials in the workplace. The National Industrial Chemicals Notification and Assessment Scheme (NICNAS) also provides guidance on the safe handling and use of nanomaterials (Safe Work Australia, 2019).
4. The Therapeutic Goods Administration (TGA) regulates the safety and efficacy of therapeutic products, including those that use nanotechnology. In 2015, the TGA released a guidance document on the regulation of medicines that contain nanomaterials (TGA, 2015).

***China***: In China, the regulation of nanotechnology-based agricultural products falls under the jurisdiction of several government agencies, including the Ministry of Agriculture and Rural Affairs (MARA), the State Administration for Market Regulation (SAMR), the National Health Commission (NHC), and the Ministry of Ecology and Environment (MEE). The following are some key laws, safety standards, and regulations related to nanotechnology-based agricultural products in China, along with their references and years of enactment (http://en.nim.ac.cn/; http://en.nim.ac.cn/division/overview/924).

1. Regulations on the Safety Assessment of Agricultural Genetically Modified Organisms (MARA Order No. 7)—2001. This regulation sets out the safety requirements and procedures for the approval of genetically modified agricultural products, including those that utilize nanotechnology.
2. Safety Requirements for Food and Food Additives Containing Nanomaterials (NHC No. 13)—2011. This guideline establishes safety requirements and evaluation procedures for food and food additives that contain nanomaterials, including those used in agriculture.
3. Technical Guidelines for Safety Assessment of Nano-Scale Agricultural Products (MARA No. 198)—2014. This guideline provides a framework for the safety assessment of nanotechnology-based agricultural products, including their production, processing, and use.
4. Administrative Measures for Safety Evaluation of New Varieties of Agricultural Genetically Modified Organisms (SAMR Order No. 8)—2020. This regulation outlines the safety evaluation procedures for new varieties of genetically modified agricultural products, including those that utilize nanotechnology.
5. Measures for the Administration of Environmental Safety Assessment of Agricultural Genetically Modified Organisms (MEE Order No. 12)—2021. This regulation sets out the procedures and requirements for the environmental safety assessment of genetically modified agricultural products, including those that use nanotechnology.

***India***: In India, the regulation of nanotechnology-based agri-products falls under the purview of various agencies and laws, including the Department of Biotechnology (DBT), the Ministry of Environment, Forest and Climate Change (MoEFCC), the Food Safety and Standards Authority of India (FSSAI), and the Indian Council of Agricultural Research (ICAR).

Here are some laws, safety measures, and regulations related to nanotechnology-based agri-products in India (dbtindia.gov.in).

1. The Environment (Protection) Act, 1986: This law empowers the MoEFCC to regulate the production, import, export, and use of hazardous substances, including nanomaterials (Environment Protection Act, 1986).
2. The Hazardous Waste (Management, Handling, and Transboundary Movement) Rules, 2016: These rules require the registration and authorization of facilities that generate, store, and dispose of hazardous wastes, including nanomaterials (Hazardous Waste Rules, 2016).
3. The Food Safety and Standards Act, 2006: This law establishes the Food Safety and Standards Authority of India (FSSAI), which regulates the safety and quality of food products in India. The FSSAI has issued guidelines for the use of nanotechnology in food products, including agri-products (Food Safety and Standards Act, 2006).
4. The Insecticides Act, 1968: This law regulates the registration, sale, distribution, and use of insecticides in India. Nanotechnology-based insecticides fall under the purview of this act (Insecticides Act, 1968).
5. The Seeds Act, 1966: This law regulates the quality of seeds used in agriculture. The act has been amended to include provisions for the regulation of genetically modified seeds, which may include the use of nanotechnology (Seeds Act, 1966).
6. DBT Guidelines on Safety Assessment of Foods Derived from Genetically Engineered Plants and Microorganisms (2017): These guidelines provide a framework for the safety assessment of foods derived from genetically engineered plants and microorganisms, including those produced using nanotechnology.
7. MoEFCC Notification on Manufacture, Storage and Import of Hazardous Chemicals Rules (1989): This notification requires manufacturers and importers of hazardous chemicals, including nanomaterials, to comply with certain safety and environmental regulations.
8. FSSAI Regulations on Food Additives (2011): These regulations specify the conditions for the use of food additives, including those derived from nanotechnology, in food products.
9. ICAR Guidelines on Nanotechnology Research in Agriculture (2010): These guidelines provide a framework for the safe and responsible use of nanotechnology in agricultural research and development.
10. Indian Pharmacopoeia Commission (IPC) Guidelines on Nanoparticle Characterization (2019): These guidelines provide a framework for the characterization of nanoparticles, including those used in the production of agri-products.

Overall, the regulation of nanotechnology-based agri-products in India is still evolving, and there is a need for more comprehensive and coordinated regulatory frameworks to ensure their safety and efficacy.

*Note*: The above laws, safety measures, and regulations related to nanotechnology-based agri-products in India may also be subject to additional guidance and policies issued by the respective regulatory agencies. It is important to note that these regulations are constantly evolving and subject to change, and there may be additional guidelines and standards at the local or regional levels.

## Conclusion and future prospects

Nanotechnology have paved a way to find out the new strategies to develop novel methods to bring scientific interventions that enabled us to raise a quality product in the field of agriculture and production of agri-products. Although, there are few ill effects of the technology which need to be mitigated to make it a successful approach. Further, researchers or scientists need to work on green synthesis approach to make it more reliable and ecofriendly which is the utmost need of the society. Green synthesis technique does not require any toxic solvent as a capping and reducing agent which eradicates the environmental pollution. Nano priming of seeds can also be the one helpful technique in order to maintain the pace of sustainable agriculture through the development of nano-primed plants that bear alterations at the molecular level and produces ultimate modifications in the phytochemicals and physiological changes in the plant without causing any harmful effect to the environment and plant itself. Moreover, it is simple, cost effective and requires less energy. Bottom down method should be focused. Apart from this, primary screening needs to be done to decide the usage of optimal dose or concentration of the chemicals or extracts used. Mode of delivery of nanoparticles should be specifically monitored or framed so that it will not carry any toxic substance with it. The effectiveness of respective regulatory systems for handling nanotechnologies has been actively investigated by a number of nations worldwide. Overall, the safety measure and regulations for nanotechnology based agri-product need to be updated time to time as the research in this field continues to come out with the new scientific interventions and its output which need to be screened by the regulatory bodies.

## References

[B1] Abdel-Razik A. Hammad I. Tawfik E. (2017). Transformation of thionin genes using chitosan nanoparticle into potato plant to be resistant to fungal infection. IOSR J. Biotechnol. Biochem. 3, 01–13. 10.9790/264x-03030113

[B2] Afsharinejad A. Davy A. Jennings B. Brennan C. (2016). Performance analysis of plant monitoring nanosensor networks at THz frequencies. IEEE Internet Things J. 3, 59–69. 10.1109/jiot.2015.2463685

[B3] Afzal S. Sharma D. Singh N. K. (2021). Eco-friendly synthesis of phytochemical-capped iron oxide nanoparticles as nano-priming agent for boosting seed germination in rice (*Oryza sativa* L). Environ. Sci. Pollut. Res. Int. 28, 40275–40287. 10.1007/s11356-020-12056-5 33447981

[B4] Ahmar S. Mahmood T. Fiaz S. Mora-Poblete F. Shafique M. S. Chattha M. S. (2021). Advantage of nanotechnology-based genome editing system and its application in crop improvement. Front. Plant Sci. 12, 663849. 10.3389/fpls.2021.663849 34122485 PMC8194497

[B5] Al-Mamun M. R. Hasan M. R. Ahommed M. S. Bacchu M. S. Ali M. R. Khan M. Z. H. (2021). Nanofertilizers towards sustainable agriculture and environment. Environ. Technol. Innovation 23, 101658. 10.1016/j.eti.2021.101658

[B6] Altman A. Hasegawa P. M. (2011). Plant biotechnology and agriculture: Prospects for the 21st century. Academic Press.

[B7] Altpeter F. Springer N. M. Bartley L. E. Blechl A. E. Brutnell T. P. Citovsky V. (2016). Advancing crop transformation in the era of genome editing. Plant Cell 28, 1510–1520. 10.1105/tpc.16.00196 27335450 PMC4981132

[B8] An C. Sun C. Li N. Huang B. Jiang J. Shen Y. (2022). Nanomaterials and nanotechnology for the delivery of agrochemicals: Strategies towards sustainable agriculture. J. Nanobiotechnology 20, 11–19. 10.1186/s12951-021-01214-7 34983545 PMC8725417

[B9] Anjali C. Khan S. S. Margulis-Goshen K. Magdassi S. Mukherjee A. Chandrasekaran N. (2010). Formulation of water-dispersible nanopermethrin for larvicidal applications. Ecotoxicol. Environ. Saf. 73, 1932–1936. 10.1016/j.ecoenv.2010.08.039 20833431

[B10] Anjum N. A. Singh N. Singh M. K. Sayeed I. Duarte A. C. Pereira E. (2014). Single-bilayer graphene oxide sheet impactsunderlying potential mechanism assessment in germinating faba bean (*Vicia faba* L). Sci. Total Environ. 472, 834–841. 10.1016/j.scitotenv.2013.11.018 24342089

[B11] APVMA (2014). Guidance on the regulation of nanomaterials in pesticides and veterinary medicines. Retrieved from https://apvma.gov.au/node/26621 .

[B12] Arya S. S. Lenka S. K. Cahill D. M. Rookes J. E. (2021b). Designer nanoparticles for plant cell culture systems: Mechanisms of elicitation and harnessing of specialized metabolites. BioEssays 43, e2100081. 10.1002/bies.202100081 34608646

[B13] Arya S. S. Mahto B. K. Ramkumar T. R. Lenka S. K. (2020). Sharpening gene editing toolbox in Arabidopsis for plants. J. Plant Biochem. Biotechnol. 29, 769–784. 10.1007/s13562-020-00606-4

[B14] Arya S. S. Rookes J. E. Cahill D. M. Lenka S. K. (2022). Chitosan nanoparticles and their combination with methyl jasmonate for the elicitation of phenolics and flavonoids in plant cell suspension cultures. Int. J. Biol. Macromol. 214, 632–641. 10.1016/j.ijbiomac.2022.06.145 35760163

[B15] Arya S. S. Tanwar N. Lenka S. K. (2021a). Prospects of nano- and peptide-carriers to deliver CRISPR cargos in plants to edit across and beyond central dogma. Nanotechnol. Environ. Eng. 6, 22. 10.1007/s41204-021-00118-z

[B16] Attia E. A. Elhawat N. (2021). Combined foliar and soil application of silica nanoparticles enhances the growth,flowering period and flower characteristics of marigold (Tageteserecta L). Sci. Hortic. 282, 110015. 10.1016/j.scienta.2021.110015

[B17] Babu S. Singh R. Yadav D. Rathore S. S. Raj R. Avasthe R. (2022). Nanofertilizers for agricultural and environmental sustainability. Chemosphere 292, 133451. 10.1016/j.chemosphere.2021.133451 34973251

[B19] Barrena R. Casals E. Colón J. Font X. Sánchez A. Puntes V. (2016). Evaluation of the ecotoxicity of model nanoparticles. Chemosphere 144, 850–857. 10.1016/j.chemosphere.2009.01.078 19264345

[B20] Bartholomaeus N. F. A. A. (2011). Regulation of nanotechnologies in food in Australia and New Zealand. Int. Food Risk Anal. J. 1 (2), 33e40. 10.5772/10685

[B21] Bates K. Kostarelos K. (2013). Carbon nanotubes as vectors for gene therapy: Past achievements, present challenges and future goals. Adv. drug Deliv. Rev. 65, 2023–2033. 10.1016/j.addr.2013.10.003 24184373

[B22] Bellingham B. K. (2011). Proximal soil sensing. Vadose Zone J. 10, 1340–1341. 10.2136/vzj2011.0105br

[B23] Burlaka O. Pirko Y. V. Yemets A. Blume Y. B. (2015). Plant genetic transformation using carbon nanotubes for DNA delivery. Cytol. Genet. 49, 349–357. 10.3103/s009545271506002x 26841488

[B24] CAPA (1985). Canada agricultural products act (CAPA) R.S.C c 20 (4th supp). Available at: https://laws-lois.justice.gc.ca/eng/acts/A-6.01/ .

[B25] CEPA (1999). Canadian environmental protection act, 1999 (CEPA) C 33). Available at: https://laws-lois.justice.gc.ca/eng/acts/C-15.31/ .

[B26] Cesarino I. Moraes F. C. Lanza M. R. V. Machado S. A. S. (2012). Electrochemical detection of carbamate pesticides in fruit and vegetables with a biosensor based on acetylcholinesterase immobilised on a composite of polyaniline–carbon nanotubes. Food Chem. 135, 873–879. 10.1016/j.foodchem.2012.04.147 22953799

[B27] Chandrasekaran R. Rajiv P. Abd-Elsalam K. A. (2020). “Carbon nanotubes: Plant gene delivery and genome editing,” in Carbon nanomaterials for agri-food and environmental applications (Elsevier), 279–296.

[B28] Chang F.-P. Kuang L.-Y. Huang C.-A. Jane W.-N. Hung Y. Yue-Ie C. H. (2013). A simple plant gene delivery system using mesoporous silica nanoparticles as carriers. J. Mater. Chem. B 1, 5279–5287. 10.1039/c3tb20529k 32263331

[B29] Chemicals and Polymers (New Substances Notification Regulations) (2015). Chemicals and polymers (new substances notification regulations) SOR/2015-81. Retrieved from https://laws-lois.justice.gc.ca/PDF/SOR-2015-81.pdf .

[B30] Chen C. P. Chou J. C. Liu B. R. Chang M. Lee H. J. (2007). Transfection and expression of plasmid DNA in plant cells by an arginine-rich intracellular delivery peptide without protoplast preparation. FEBS Lett. 581, 1891–1897. 10.1016/j.febslet.2007.03.076 17433309

[B31] Chen H. Yada R. Chang Y. Bu R. Li Y. h. Liu M. (2011). Nanotechnologies in agriculture: New tools for sustainable development. Trends Food Sci. Technol. 22, 585–594. 10.1016/j.tifs.2011.09.004

[B32] Chhipa H. Maheshwari S. Upadhyay R. Upadhyay B. Sain H. (2019). Nanotechnology in agriculture: A review. Int. J. Curr. Microbiol. Appl. Sci. 8 (1), 2377–2383. 10.20546/ijcmas.2019.805.281

[B33] Christian M. Cermak T. Doyle E. L. Schmidt C. Zhang F. Hummel A. (2010). Targeting DNA double-strand breaks with TAL effector nucleases. Genetics 186, 757–761. 10.1534/genetics.110.120717 20660643 PMC2942870

[B34] Chuah J. A. Yoshizumi T. Kodama Y. Numata K. (2015). Gene introduction into the mitochondria of *Arabidopsis thaliana* via peptide-based carriers. Sci. Rep. 5, 7751. 10.1038/srep07751 25583214 PMC4291575

[B35] Clement L. Hurel C. Marmier N. (2013). Toxicity of TiO(2) nanoparticles to cladocerans, algae, rotifers and plants - effects of size and crystalline structure. Chemosphere 90 (I.3), 1083–1090. 10.1016/j.chemosphere.2012.09.013 23062945

[B36] Cunningham F. J. Goh N. S. Demirer G. S. Matos J. L. Landry M. P. (2018). Nanoparticle-mediated delivery towards advancing plant genetic engineering. Trends Biotechnol. 36, 882–897. 10.1016/j.tibtech.2018.03.009 29703583 PMC10461776

[B37] Demirer G. S. Landry M. P. (2017). Delivering genes to plants. Chem. Eng. Prog. 113, 40–45.

[B38] Demirer G. S. Zhang H. Goh N. S. González-Grandío E. Landry M. P. (2019). Carbon nanotube–mediated DNA delivery without transgene integration in intact plants. Nat. Protoc. 14, 2954–2971. 10.1038/s41596-019-0208-9 31534231 PMC10496593

[B40] Dimkpa C. O. Bindraban P. S. (2017). Nanofertilizers: New products for the industry? J. Agric. food Chem. 66, 6462–6473. 10.1021/acs.jafc.7b02150 28535672

[B41] DST (2018). Department of science and technology, nanotechnology. Retrieved from http://www.dst.gov.in/nano-science-nanotechnology-nsnt .

[B42] EC (2018). European commission, nanotechnology. Retrieved from https://ec.europa.eu/growth/sectors/nanotechnology_en .

[B43] ECHA (2021). European chemicals agency, nanomaterials. Retrieved from https://echa.europa.eu/regulations/nanomaterials .

[B44] ECHA (2012). Updated guidance on information requirements and chemical safety assessment for nanomaterials from. Available at: http://echa.europa.eu/en/view-article/-/journal_content/3df5b7b9-a36d-4e74-811b-3aeee23366f8 .

[B45] EFSA (2018). European food safety authority, nanotechnology. Retrieved from https://www.efsa.europa.eu/en/topics/topic/nanotechnology .

[B46] EFSA Scientific Committee (2011). Guidance on the risk assessment of the application of nanoscience and nanotechnologies in the food and feed chain from. Available at: http://www.efsa.europa.eu/en/efsajournal/doc/2140.pdf . 10.2903/j.efsa.2018.5327 PMC7009542 32625968

[B47] El-Naggar M. E. Abdelsalam N. R. Fouda M. M. G. Mackled M. I. Al-Jaddadi M. A. M. Ali H. M. (2020). Soil application of nano silica on maize yield and its insecticidal activity against some stored insects after the post-harvest. Nanomaterials 10, 739. 10.3390/nano10040739 32290620 PMC7221732

[B48] Elek N. Hoffman R. Raviv U. Resh R. Ishaaya I. Magdassi S. (2010). Novaluron nanoparticles: Formation and potential use in controlling agricultural insect pests. Eng. Asp. 372, 66–72. 10.1016/j.colsurfa.2010.09.034

[B49] Environment Protection, Act (1986). Enacted in 1986 and last amended in 2015.

[B50] EPA (2017b). Quantum dots. Retrieved from https://www.epa.gov/chemical-research/quantum-dots .

[B51] EPA (2018). Silver nanoparticles. Retrieved from https://www.epa.gov/chemical-research/silver-nanoparticles .

[B52] EPA (2017a). U.S. Environmental protection agency, nanotechnology. Retrieved from https://www.epa.gov/nanotechnology .

[B53] FDA (2019b). Nanosilver. Retrieved from https://www.fda.gov/medical-devices/products-and-medical-procedures/nanosilver .

[B54] FDA (2019a). Titanium dioxide. Retrieved from https://www.fda.gov/cosmetics/cosmetic-ingredients/titanium-dioxide .

[B55] FDA (2018). U.S. Food and drug administration, nanotechnology. Retrieved from https://www.fda.gov/science-research/nanotechnology-programs-fda .

[B56] Fiaz S. Khan S. A. Anis G. B. Gaballah M. M. Riaz A. (2021). “CRISPR/Cas techniques: A new method for RNA interference in cereals,” in CRISPR and RNAi systems (Elsevier), 233–252.

[B57] Food Safety and Standards Act (2006). Enacted in 2006 and last amended in 2020.

[B58] Fortuni B. Inose T. Ricci M. Fujita Y. Van Zundert I. Masuhara A. (2019). Polymeric engineering of nanoparticles for highly efficient multifunctional drug delivery systems. Sci. Rep. 9, 2666. 10.1038/s41598-019-39107-3 30804375 PMC6389875

[B59] Fraceto L. F. Grillo R. de Medeiros G. A. Scognamiglio V. Rea G. Bartolucci C. (2016). Nanotechnology in agriculture: Which innovation potential does it have. Front. Environ. Sci. 4, 20. 10.3389/fenvs.2016.00020

[B60] Frederiksen H. K. Kristensen H. G. Pedersen M. (2003). Solid lipid microparticle formulations of the pyrethroid gamma-cyhalothrin—Incompatibility of the lipid and the pyrethroid and biological properties of the formulations. J. Control. release 86, 243–252. 10.1016/s0168-3659(02)00406-6 12526821

[B61] FSANZ (2015). Titanium dioxide nanoparticles in food. Retrieved from https://www.foodstandards.gov.au/publications/Documents/Titanium-dioxide-nanoparticles-in-food-information-paper.pdf .

[B62] FSSAI (2018). Food safety and standards authority of India, nanotechnology. Retrieved from https://www.fssai.gov.in/cms/nanotechnology.php .

[B63] Guan H. Chi D. Yu J. Li X. (2008). A novel photodegradable insecticide: Preparation, characterization and properties evaluation of nano-Imidacloprid. Pesticide Biochem. Physiology 92, 83–91. 10.1016/j.pestbp.2008.06.008

[B64] Guillette J. Louis J. Iguchi T. (2012). Ecology. Life in a contaminated world. Science 337, 1614–1615. 10.1126/science.1226985 23019638

[B65] Hajiahmadi Z. Shirzadian-Khorramabad R. Kazemzad M. Sohani M. M. (2019). Enhancement of tomato resistance to Tuta absoluta using a new efficient mesoporous silica nanoparticle-mediated plant transient gene expression approach. Sci. Hortic. 243, 367–375. 10.1016/j.scienta.2018.08.040

[B66] Haris M. Hussain T. Mohamed H. I. Khan A. Ansari M. S. Tauseef A. (2023). Nanotechnology – a new frontier of nano-farming in agricultural and food production and its development. Sci. Total Environ. 857, 159639. 10.1016/j.scitotenv.2022.159639 36283520

[B67] Hassani A. Ahmad M. B. Abdullah A. H. Abdul Rahman M. B. Shameli K. (2020). Nanoparticles and nanomaterials: An overview of their biological effects on plants. J. Nanomater. 2020.

[B68] Hazardous Waste (Management, Handling, and Transboundary Movement) Rules (2016). Enacted in 2016.

[B69] He X. Deng H. Hwang H.-M. (2019). The current application of nanotechnology in food and agriculture. J. food drug analysis 27, 1–21. 10.1016/j.jfda.2018.12.002 PMC9298627 30648562

[B70] Huang L. Li X. Chen M. Chen L. (2019). Advances in genetic engineering of crops for abiotic stress tolerance. J. Agric. Food Chem. 67 (48), 13127–13140.31682438

[B71] Huang S. Wang L. Liu L. Hou Y. Li L. (2015). Nanotechnology in agriculture, livestock, and aquaculture in China. A review. Agron. Sustain. Dev. 35, 369–400. 10.1007/s13593-014-0274-x

[B72] Imtiaz H. Shiraz M. Mir A. R. Siddiqui H. Hayat S. (2023). Nano-priming techniques for plant physio-biochemistry and stress tolerance. J. Plant Growth Regul. 10.1007/s00344-023-10981-6

[B73] Insecticides Act (1968). Enacted in 1968 and last amended in 2000.

[B74] Jalali M. Ghanati F. Modarres-Sanavi A. M. (2016). Effect of Fe3O4 nanoparticles and iron chelate on the antioxidant capacity and nutritional value of soilcultivated maize (*Zea mays*) plants. Crop Pasture Sci. 67, 621–628. 10.1071/cp15271

[B75] Jiang L. Ding L. He B. Shen J. Xu Z. Yin M. (2014). Systemic gene silencing in plants triggered by fluorescent nanoparticle-delivered double-stranded RNA. Nanoscale 6, 9965–9969. 10.1039/c4nr03481c 25068243

[B76] Jinek M. Chylinski K. Fonfara I. Hauer M. Doudna J. A. Charpentier E. (2012). A programmable dual-RNA-guided DNA endonuclease in adaptive bacterial immunity. Science 337, 816–821. 10.1126/science.1225829 22745249 PMC6286148

[B77] Kafshgari M. H. Delalat B. Tong W. Y. Harding F. J. Kaasalainen M. Salonen J. (2015). Oligonucleotide delivery by chitosan-functionalized porous silicon nanoparticles. Nano Res. 8, 2033–2046. 10.1007/s12274-015-0715-0

[B78] Kah M. Beulke S. Tiede K. Hofmann T. (2013). Nanopesticides: State of knowledge, environmental fate, and exposure modeling. Critical Reviews in Environmental Science and Technology.

[B79] Kah M. Tufenkji N. White J. C. (2019). Nano-enabled strategies to enhance crop nutrition and protection. Nat. Nanotechnol. 14, 532–540. 10.1038/s41565-019-0439-5 31168071

[B80] Kang B. C. Bae S. J. Lee S. Lee J. S. Kim A. Lee H. (2021). Chloroplast and mitochondrial DNA editing in plants. Nat. Plants 7, 899–905. 10.1038/s41477-021-00943-9 34211132 PMC8289734

[B81] Karimi M. Ghasemi A. Zangabad P. S. Rahighi R. Basri S. M. M. Mirshekari H. (2016). Smart micro/nanoparticles in stimulus-responsive drug/gene delivery systems. Chem. Soc. Rev. 45, 1457–1501. 10.1039/c5cs00798d 26776487 PMC4775468

[B82] Khalifa N. S. Hasaneen M. N. (2018). The effect of chitosan–PMAA–NPK nanofertilizer on Pisum sativum plants. 3 Biotech. 8, 193. 10.1007/s13205-018-1221-3 PMC5861260 29576999

[B83] Khodakovskaya M. V. Kim B. S. Kim J. N. Alimohammadi M. Dervishi E. Mustafa T. (2013). Carbon nanotubes as plant growth regulators: Effects on tomato growth, reproductive system, and soil microbial community. Small 9, 115–123. 10.1002/smll.201201225 23019062

[B84] Khot L. R. Sankaran S. Maja J. M. Ehsani R. Schuster E. W. (2020). Applications of nanomaterials in agricultural production and crop protection: A review. Crop Prot. 133, 105221. 10.1016/j.cropro.2012.01.007

[B85] Kookana R. S. Boxall A. B. Reeves P. T. Ashauer R. Beulke S. Chaudhry Q. (2014). Nanopesticides: Guiding principles for regulatory evaluation of environmental risks. J. Agric. Food Chem. 62 (19), 4227–4240. 10.1021/jf500232f 24754346

[B86] Kookana R. S. Sarmah A. K. Van Z. L. Krull E. Singh B. (2011). Biochar application to soil: Agronomic and environmental benefits and unintended consequences. Adv. Agron. 112, 103–143. 10.1016/B978-0-12-385538-1.00003-2

[B87] Kuzma J. Kokotovich A. (2011). Renegotiating GM crop regulation. Targeted gene-modification technology raises new issues for the oversight of genetically modified crops. EMBO Rep. 12 (9), 883–888. 10.1038/embor.2011.160 21836639 PMC3166464

[B88] Lander E. S. (2016). The heroes of CRISPR. Cell 164 (1-2), 18–28. 10.1016/j.cell.2015.12.041 26771483

[B89] Liu D. Chen W. Wei J. Li X. Wang Z. Jiang X. (2012). A highly sensitive, dual-readout assay based on gold nanoparticles for organophosphorus and carbamate pesticides. Anal. Chem. 84, 4185–4191. 10.1021/ac300545p 22475016

[B90] Liu Q. Zhang X. Zhao Y. Lin J. Shu C. Wang C. (2013). Fullerene-Induced increase of glycosyl residue on living plant cell wall. Environ. Sci. Technol. 47, 7490–7498. 10.1021/es4010224 23721301

[B91] Liu Q. Zhao Y. Wan Y. Zheng J. Zhang X. Wang C. (2010). Study of the inhibitory effect of water-soluble fullerenes on plant growth at the cellular level. ACS Nano 4 (10), 5743–5748. 10.1021/nn101430g 20925388

[B92] Liu R. Lal R. Lin Z. (2016). Effects of nanomaterials on soil properties and crop growth. J. Nanoparticle Res. 18 (9), 262.

[B93] López-Vargas E. R. Ortega-Ortíz H. Cadenas-Pliego G. Romenus K. D. A. de la Fuente M. C. Benavides-Mendoza A. (2018). Foliar application of copper nanoparticles increases the fruit quality and the content of bioactive compounds in tomatoes. Appl. Sci. 8, 1020. 10.3390/app8071020

[B94] Ma X. Geiser-Lee J. Deng Y. Kolmakov A. (2010). Interactions between engineered nanoparticles (ENPs) and plants: Phytotoxicity, uptake and accumulation. Sci. Total. Environ. 408, 3053–3061. 10.1016/j.scitotenv.2010.03.031 20435342

[B95] Mahakham W. Sarmah A. K. Maensiri S. Theerakulpisut P. (2017). Nanopriming technology for enhancing germination and starch metabolism of aged rice seeds using phytosynthesized silver nanoparticles. Sci. Rep. 7, 8263. 10.1038/s41598-017-08669-5 28811584 PMC5557806

[B96] Mahto B. K. Singh A. Pareek M. Rajam M. V. Dhar-Ray S. Reddy P. M. (2020). Host-induced silencing of the Colletotrichum gloeosporioides conidial morphology 1 gene (CgCOM1) confers resistance against Anthracnose disease in chilli and tomato. Plant Mol. Biol. 104, 381–395. 10.1007/s11103-020-01046-3 32803478

[B97] MARA (2017). Ministry of agriculture and rural Affairs, nanotechnology. Retrieved from http://www.moa.gov.cn/english/nature/standards/nanotech/index.htm .

[B98] Mishra A. Singh H. B. (2021). “Nanoparticles in agro-products: Benefits, applications and concerns,” in Nanomaterials for agriculture and forestry applications (Singapore: Springer), 73–88.

[B99] Miyamoto T. Tsuchiya K. Numata K. (2018). Block copolymer/plasmid DNA micelles postmodified with functional peptides via thiol–maleimide conjugation for efficient gene delivery into plants. Biomacromolecules 20, 653–661. 10.1021/acs.biomac.8b01304 30257560

[B100] Miyamoto T. Tsuchiya K. Numata K. (2020). Dual peptide-based gene delivery system for the efficient transfection of plant callus cells. Biomacromolecules 21, 2735–2744. 10.1021/acs.biomac.0c00481 32432860

[B101] MoEFCC (2014). Ministry of environment, forest and climate change, nanotechnology. Retrieved from http://www.moef.gov.in/nanotechnology .

[B102] Moon H. Lee J. Min J. Kang S. (2014). Developing genetically engineered encapsulin protein cage nanoparticles as a targeted delivery nanoplatform. Biomacromolecules 15, 3794–3801. 10.1021/bm501066m 25180761

[B103] Mukhopadhyay S. S. (2014). Nanotechnology in agriculture: Prospects and constraints. Nanotechnol. Sci. Appl. 7, 63–71. 10.2147/NSA.S39409 25187699 PMC4130717

[B104] Mura S. Greppi G. Roggero P. P. Musu E. Pittalis D. Carletti A. (2015). Functionalized gold nanoparticles for the detection of nitrates in water. Int. J. Environ. Sci. Technol. 12, 1021–1028. 10.1007/s13762-013-0494-7

[B107] Nel A. Xia T. Mädler L. Li N. (2006). Toxic potential of materials at the nanolevel. Science 311, 622–627. 10.1126/science.1114397 16456071

[B108] NIOSH (2013). Carbon nanotubes. Retrieved from https://www.cdc.gov/niosh/topics/nanotech/pdfs/CarbonNanotubes2009.pdf .

[B109] NNSC (2017). National nanotechnology standardization technical committee, nanotechnology. Retrieved from http://www.nssc.org.cn/english/ .

[B110] Oliveira H. C. Gomes B. C. R. Pelegrino M. T. Seabra A. B. (2016). Nitric oxide-releasing chitosan nanoparticles alleviate the effects of salt stress in maize plants. Nitric Oxide 61, 10–19. 10.1016/j.niox.2016.09.010 27693703

[B111] Oliveira H. C. Stolf-Moreira R. Martinez C. B. R. Grillo R. De Jesus M. B. Fraceto L. F. (2015). Nanoencapsulation enhances the post-emergence herbicidal activity of atrazine against mustard plants. PLoS One 10, e0132971. 10.1371/journal.pone.0132971 26186597 PMC4506088

[B112] Pan W. Tao J. Cheng T. Bian X. Wei W. Zhang W. (2016). Soybean *miR172a* improves salt tolerance and can function as a long-distance signal. Mol. Plant 9, 1337–1340. 10.1016/j.molp.2016.05.010 27235547

[B113] Parisi C. Vigani M. Rodríguez-Cerezo E. (2015). Agricultural nanotechnologies: What are the current possibilities? Nano Today 10, 124–127. 10.1016/j.nantod.2014.09.009

[B114] Pauwels K. van der Straeten D. (2016). “Ethical issues in genetic engineering and transgenics,” in Reference module in food science (Elsevier), 1–6.

[B115] PCPA (2002). Pest control products act (PCPA) (S C 2002, c 28). Available at: https://laws-lois.justice.gc.ca/eng/acts/P-9.01/ .

[B116] Pereira A. E. S. Sandoval-Herrera I. E. Zavala-Betancourt S. A. Oliveira H. C. Ledezma-Pérez A. S. Romero J. (2017). γ-Polyglutamic acid/chitosan nanoparticles for the plant growth regulator gibberellic acid: Characterization and evaluation of biological activity. Carbohydr. Polym. 157, 1862–1873. 10.1016/j.carbpol.2016.11.073 27987906

[B117] Powell J. J. Ainley K. Harvey R. S. Mason A. S. (2016). The role of oral processing in nanoparticle uptake and relevance to consumer exposure: A review. Br. J. Nutr. 116 (11), 1886–1897.

[B118] Pramanik S. Ray S. Das M. Bhattacharjee R. Mukherjee B. Mondal S. A. (2020). Nanotechnology in agriculture: Challenges and opportunities. J. Exp. Biol. Agric. Sci. 8 (1), 160–164. 10.4103/ijem.IJEM_561_19

[B149] Prasad R. Kumar V. Prasad K. S. (2014). Nanotechnology in sustainable agriculture: present concerns and future aspects. Afr. J. Biotechnol. 13 (6), 705–713. 10.5897/AJBX2013.13554

[B150] Prasad R. Bhattacharyya A. Nguyen Q. D. (2017). Nanotechnology in sustainable agriculture: Recent developments, challenges, and perspectives. Front. Microbiol. 8, 1014. 10.3389/fmicb.2017.01014 28676790 PMC5476687

[B119] Puchta H. Dujon B. Hohn B. (1993). Homologous recombination in plant cells is enhanced by *in vivo* induction of double strand breaks into DNA by a site-specific endonuclease. Nucleic acids Res. 21, 5034–5040. 10.1093/nar/21.22.5034 8255757 PMC310614

[B120] Pullagurala V. L. R. Adisa I. O. Rawat S. Kalagara S. Hernandez-Viezcas J. A. Peralta-Videa J. R. (2018). ZnO nanoparticles increase photosynthetic pigments and decrease lipid peroxidation in soil grown cilantro (*Coriandrum sativum)* . Plant physiology Biochem. 132, 120–127. 10.1016/j.plaphy.2018.08.037 30189415

[B121] Purnell M. Wong W. Kado T. (2018). International governance of genetically modified crops: A global environmental and human health risk analysis. Environ. Sci. Policy 84, 60–69.

[B122] Rafsanjani M. Kiran U. Ali A. Abdin M. (2016). Transformation efficiency of calcium phosphate nanoparticles for genetic manipulation of *Cichorium intybus* L.

[B123] Rajput V. Minkina T. Sushkova S. Behal A. Maksimov A. Blicharska E. (2020). ZnO and CuO nanoparticles: A threat to soil organisms, plants, and human health. Environ. Geochem. Health 42 (1), 147–158. 10.1007/s10653-019-00317-3 31111333

[B124] Rico C. M. Majumdar S. Duarte-Gardea M. Peralta-Videa J. R. Gardea- Torresdey J. L. (2011). Interaction of nanoparticles with edible plants and their possible implications in the food chain. J. Agricul. Food. Chem. 59, 3485–3498. 10.1021/jf104517j PMC3086136 21405020

[B125] Rosenzweig C. Mbow C. Barioni L. G. Benton T. G. Herrero M. Krishnapillai M. (2020). Climate change responses benefit from a global food system approach. Nat. Food 1, 94–97. 10.1038/s43016-020-0031-z 37128000

[B126] Safe Work Australia (2019). Safe work Australia nanotechnology. Retrieved from https://www.safeworkaustralia.gov.au/topic/nanotechnology .

[B127] Samboju N. C. Yau K. Y. Burroughs F. G. Samuel J. P. Webb S. R. (2012). Linear DNA molecule delivery using PEGylated quantum dots for stable transformation in plants. Google Patents.

[B128] SAMR (2018). State administration for market regulation, guidelines for the safety assessment and management of nanomaterials. Retrieved from http://gkml.samr.gov.cn/nsjg/ggtg/201812/t20181204_291576.html .

[B129] Samuel J. P. Petolino J. F. Samboju N. C. Webb S. R. Yau K. Y. (2015). Nanoparticle mediated delivery of sequence specific nucleases. Google Patents.

[B130] Sashidhar P. Arya S. S. Das R. K. Dubey M. K. Lenka S. K. (2021). “Nanobiotechnology for plant genome engineering and crop protection,” in Genetically modified crops in asia pacific, 279–310.

[B131] Sashidhar P. Kochar M. Singh B. Gupta M. Cahill D. Adholeya A. (2020). Biochar for delivery of agri-inputs: Current status and future perspectives. Sci. Total Environ. 703, 134892. 10.1016/j.scitotenv.2019.134892 31767299

[B132] Sashidhar R. B. Dubey M. K. Kochar M. (2019). “Sensing soil microbes and interactions: How can nanomaterials help?,” in Microbial nanobionics. Nanotechnology in the life sciences. Editor Prasad R. (Cham: Springer). 10.1007/978-3-030-16534-5_11

[B133] Saxena M. Maity S. Sarkar S. (2014). Carbon nanoparticles in ‘biochar’boost wheat (*Triticum aestivum*) plant growth. Rsc Adv. 4, 39948–39954. 10.1039/c4ra06535b

[B134] Seeds Act (1966). Enacted in 1966 and last amended in 2004.

[B135] Shah T. Latif S. Saeed F. Ali I. Ullah S. Abdullah Alsahli A. (2021). Seed priming with titanium dioxide nanoparticles enhances seed vigor, leaf water status, and antioxidant enzyme activities in maize (*Zea mays* L) under salinity stress. J. King Saud. Univ. Sci. 33, 101207. 10.1016/j.jksus.2020.10.004

[B136] Shaheen A. Abed Y. (2018). Knowledge, attitude, and practice among farmworkers applying pesticides in cultivated area of the jericho district: A cross-sectional study. Lancet 391, S3. 10.1016/s0140-6736(18)30328-3 29553428

[B137] Silva A. T. Nguyen A. Ye C. Verchot J. Moon J. H. (2010). Conjugated polymer nanoparticles for effective siRNA delivery to tobacco BY-2 protoplasts. BMC plant Biol. 10, 291–314. 10.1186/1471-2229-10-291 21192827 PMC3023792

[B138] Singh S. Tripathi D. K. Singh S. Sharma S. Dubey N. K. Chauhan D. K. (2021). Nano-based approach to enhance crop productivity under changing climate: Present status and future prospects. J. Clean. Prod. 286, 125605.

[B139] Sonawane H. Arya S. Math S. Shelke D. (2021). Myco-synthesized silver and titanium oxide nanoparticles as seed priming agents to promote seed germination and seedling growth of *Solanum lycopersicum*: A comparative study. Int. Nano Lett. 11, 371–379. 10.1007/s40089-021-00346-w

[B140] Soni A. T. James E. R. Arya S. S. (2023). Chitosan nanoparticles as seed priming agents to alleviate salinity stress in rice (*Oryza sativa* L) seedlings. Polysaccharides 4 (2), 129–141. 10.3390/polysaccharides4020010

[B141] Su Y. Wu D. Xia H. Zhang C. Shi J. Wilkinson K. J. (2019). Metallic nanoparticles induced antibiotic resistance genes attenuation of leachate culturable microbiota: The combined roles of growth inhibition, ion dissolution and oxidative stress. Environ. Int. 128, 407–416. 10.1016/j.envint.2019.05.007 31078875

[B142] Sun J. K. Honglu W. Dong-Il M. Myeong-Lok S. Beomseok K. Dong I. L. (2019). Carbon nanotube based γ ray detector. ACS Sensors 4 (4), 1097–1102. 10.1021/acssensors.9b00380 30848593

[B143] Talarico D. Arduini F. Amine A. Cacciotti I. Moscone D. Palleschi G. (2016). Screen-printed electrode modified with carbon black and chitosan: A novel platform for acetylcholinesterase biosensor development. Anal. Bioanal. Chem. 408, 7299–7309. 10.1007/s00216-016-9604-y 27251198

[B144] Tarafdar J. C. Raliya R. Mahawar H. Rathore I. (2014). Development of zinc nanofertilizer to enhance crop production in pearl millet (*Pennisetum americanum*). Agric. Res. 3, 257–262. 10.1007/s40003-014-0113-y

[B145] Telarico L. Georgiev V. (2016). Modelli di Kuramoto non abeliani ed approccio quantistico alle reti neurali.

[B146] Terada K. Gimenez-Dejoz J. Miyagi Y. Oikawa K. Tsuchiya K. Numata K. (2020). Artificial cell-penetrating peptide containing periodic α-aminoisobutyric acid with long-term internalization efficiency in human and plant cells. ACS Biomaterials Sci. Eng. 6, 3287–3298. 10.1021/acsbiomaterials.0c00182 33463179

[B147] TGA (2015). Regulation of therapeutic goods containing nanomaterials. Retrieved from https://www.tga.gov.au/publication/regulation-therapeutic-goods-containing-nanomaterials .

[B148] Thagun C. Motoda Y. Kigawa T. Kodama Y. Numata K. (2020). Simultaneous introduction of multiple biomacromolecules into plant cells using a cell-penetrating peptide nanocarrier. Nanoscale 12, 18844–18856. 10.1039/d0nr04718j 32896843

[B151] Tripathi K. M. Bhati A. Singh A. Sonker A. K. Sarkar S. Sonkar S. K. (2017). Sustainable changes in the contents of metallic micronutrients in first generation gram seeds imposed by carbon nano-onions: Life cycle seed to seed study. Acs Sustain. Chem. Eng. 5, 2906–2916. 10.1021/acssuschemeng.6b01937

[B152] Unnamalai N. Kang B. G. Lee W. S. (2004). Cationic oligopeptide-mediated delivery of dsRNA for post-transcriptional gene silencing in plant cells. FEBS Lett. 566, 307–310. 10.1016/j.febslet.2004.04.018 15147914

[B153] US Food and Drug Administration (2020). Cfr - code of federal regulations title 21. Retrieved from https://www.accessdata.fda.gov/scripts/cdrh/cfdocs/cfcfr/CFRSearch.cfm?fr=172.480 .

[B154] USDA (2016). U.S. Department of agriculture, nanotechnology. Retrieved from https://www.usda.gov/topics/biotechnology/nanotechnology .

[B155] U.S. Food and Drug Administration (2018). Considering whether an FDA-regulated product involves the application of nanotechnology. Guidance for industry. Available at: https://www.fda.gov/media/113994/download .

[B156] Usman M. Farooq M. Wakeel A. Nawaz A. Cheema S. A. Ur Rehman H. (2020). Nanotechnology in agriculture: Current status, challenges and future opportunities. Sci. Total Environ. 721, 137778. 10.1016/j.scitotenv.2020.137778 32179352

[B157] Wang J. W. Grandio E. G. Newkirk G. M. Demirer G. S. Butrus S. Giraldo J. P. (2019b). Nanoparticle-mediated genetic engineering of plants. Mol. plant 12, 930 1037–1040. 10.1016/j.molp.2019.06.010 PMC10461873 31283997

[B158] Wang Q. Zhang P. Zhao W. Noman S. Muhammad A. Zhu G. (2023). Effects and the fate of metal-based engineered nanomaterials on soil ecosystem: A review. Pedosphere. 10.1016/j.pedsph.2023.05.004

[B159] Wang X. Li Q. Pei Z. Zhuang J. Zhang J. Zhou G. (2019). The application of nanotechnology in agriculture: Current status and future prospects. Appl. Sci. 9 (8), 1715–1723. 10.19540/j.cnki.cjcmm.20181226.022

[B160] Wang Y. Cui H. Li K. Sun C. Du W. Cui J. (2014). A magnetic nanoparticle-based multiple-gene delivery system for transfection of porcine kidney cells. PloS one 9, e102886. 10.1371/journal.pone.0102886 25048709 PMC4105564

[B161] Wang Y. Deng C. Cota-Ruiz K. Peralta-Videa J. R. Sun Y. Rawat S. (2020). Improvement of nutrient elements and allicin content in green onion (*Allium fistulosum*) plants exposed to CuO nanoparticles. Sci. Total Environ. 725, 138387. 10.1016/j.scitotenv.2020.138387 32298898

[B162] Wang Z. Li H. Li X. Xin C. Si J. Li S. (2019a). Nano-ZnO priming induces salt tolerance by promoting photosynthetic carbon assimilation in wheat. Arch. Agron. Soil Sci. 66, 1259–1273. 10.1080/03650340.2019.1663508

[B163] Watson A. Ghosh S. Williams M. J. Cuddy W. S. Simmonds J. Rey M.-D. (2018). Speed breeding is a powerful tool to accelerate crop research and breeding. Nat. plants 4, 23–29. 10.1038/s41477-017-0083-8 29292376

[B164] Wright D. A. Townsend J. A. Winfrey R. J. Jr Irwin P. A. Rajagopal J. Lonosky P. M. (2005). High‐frequency homologous recombination in plants mediated by zinc‐finger nucleases. Plant J. 44, 693–705. 10.1111/j.1365-313X.2005.02551.x 16262717

[B165] Xu C. Cao L. Zhao P. Zhou Z. Cao C. Li F. (2018). Emulsion-based synchronous pesticide encapsulation and surface modification of mesoporous silica nanoparticles with carboxymethyl chitosan for controlled azoxystrobin release. Chem. Eng. J. 348, 244–254. 10.1016/j.cej.2018.05.008

[B166] Yan L. Qijun Z. Yongjun D. Kuizhong S. Kaimeng X. Yongchao Y. (2021). Fabricating nanodiamonds from biomass by direct laser writing under ambient conditions. ACS Sustain. Chem. Eng. 9 (8), 3112–3123. 10.1021/acssuschemeng.0c07607

[B167] Zhang H. Demirer G. S. Zhang H. Ye T. Goh N. S. Aditham A. J. (2019a). DNA nanostructures coordinate gene silencing in mature plants. Proc. Natl. Acad. Sci. 116, 7543–7548. 10.1073/pnas.1818290116 30910954 PMC6462094

[B168] Zhang Y. Chen W. Jing M. Liu S. Feng J. Wu H. (2019b). Self-assembled mixed micelle loaded with natural pyrethrins as an intelligent nano-insecticide with a novel temperature-responsive release mode. Chem. Eng. J. 361, 1381–1391. 10.1016/j.cej.2018.10.132

[B169] Zhao L. Peralta-Videa J. R. Rico C. M. Hernandez-Viezcas J. A. Sun Y. Niu G. (2014). CeO₂ and ZnO nanoparticles change the nutritional qualities of cucumber (Cucumis sativus). J. Agric. Food Chem. 62 (13), 2752–2759. 10.1021/jf405476u 24611936

[B170] Zhao X. Meng Z. Wang Y. Chen W. Sun C. Cui B. (2017). Pollen magnetofection for genetic modification with magnetic nanoparticles as gene carriers. Nat. plants 3, 956–964. 10.1038/s41477-017-0063-z 29180813

[B171] Zheng Z. Li X. Dai Z. Liu S. Tang Z. (2011). Detection of mixed organophosphorus pesticides in real samples using quantum dots/bi-enzyme assembly multilayers. J. Mater. Chem. 21, 16955–16962. 10.1039/c1jm11631b

[B172] Zhou Y. Quan G. Wu Q. Zhang X. Niu B. Wu B. (2018). Mesoporous silica nanoparticles for drug and gene delivery. Acta Pharm. sin. B 8, 165–177. 10.1016/j.apsb.2018.01.007 29719777 PMC5926503

