# Supplementary material for: Regulation and safety measures for nanotechnology-based agri-products
Source: Front Genome Ed. 2023 Jun 21;5:1200987. doi: 10.3389/fgeed.2023.1200987 (PMC10320728; doi:10.3389/fgeed.2023.1200987)
Supplement: Supplementary file 1 [file Table1.DOCX]

**Table 1**. List of NC- and PC-assisted biomolecule (DNA/RNA/protein/RNP) deliver in plant cell/tissue/organelle.

| **Plants** | **Nanocarriers (NC)/ Peptide Carrier (PC)** | **Biomolecule** | **Process** | **Targeted Cell/ Tissue/ Organelle** | **Remarks** | **References** |
| --- | --- | --- | --- | --- | --- | --- |
| **Nanocarrier based delivery** | | | | | | |
| *N. tabacum* | Fluorescent conjugated  polymer NC | RNA | Co-culture | Protoplasts | High uptake and Translocation efficiency | Silva et al., 2010 |
| *Arabidopsis thaliana* | Quantum dots  TOPO (tri-octyl phosphine  oxide)-coated CdSe/ZnS  quantum dots and polyethylene  glycol | DNA | Floral dip | Floral buds | Ease of uptake and translocation | Samboju et al., 2012 |
|  | MSNs doped with fluorescein | DNA | Co-culture | Root | High cargo capacity, surface area penetration | Chang et al., 2013 |
|  | Perylene3,4,9,10  Tetracarboxydiimide fluorescent NC | RNA | Root tip | Root | High uptake and translocation efficiency | Jiang et al., 2014 |
| *Daucus*; *Brassica* *napus* and *Glycine* *max* | Luminescent semiconductor  nanocrystals (TOPO—  CdSe/ ZnS core–shell QDs) | Protein | Co-culture | Roots and leaves | Efficient uptake in less time | Samuel et al., 2015 |
| *Cichorium intybus* | Calcium phosphate NC | DNA | Tissue dip | Leaves | A large surface area and rapid absorption | Rafsanjani et al., 2016 |
| *N. benthamiana* | Pristine carbon nanotubes; Carbon nanotubes | DNA; RNA | Co-culture; Infiltration | Leaves and protoplasts | High effectiveness of penetration and uptake | Landry et al., 2017 |
| *Solanum tuberosum* | Polymeric NC  Chitosan NC | DNA | Tissue dip | Leaves and flowers | High penetration in less time | Abdel-Razik et al., 2017 |
| Tomato | Mesoporous silica NC (MSN) | DNA | Spray and Injection | Leaves and shoots | High cargo capacity, surface area, uptake, and systemic translocation | Hajiahmadi et al., 2019 |
| **Peptide Carrier based delivery** | | | | | | |
| *A. thaliana* | CPP-MTPKH (mitochondrial targeting peptide) fused to polycationic copolymer of alternating lysine and histidine residue | DNA | Infiltration | Leaves | Effective mitochondrial localization | Unnamalai et al., 2004 |
| *N. benthamiana* | (MAL-TEG-PLL)-(Cys-NLS)-(BP100-Cys) | DNA | Infiltration | Leaves | Endocytic uptake effectively internalises cells within minutes | Chen et al., 2007 |
| *Vigna radiata* and  *Glycine max* | AID  arginine-rich intracellular delivery peptide | DNA | Co-culture | Roots | Efficient uptake by root cells | Chuah et al., 2015 |
| *A. thaliana* and  *O. sativa* | Synthetic peptide | DNA | Co-culture | Callus suspension cells | Effective translocation and internalisation through endosome | Miyamoto et al., 2018 |
| *A. thaliana* | Cytcox-KH; AtOEP34-FLAG | DNA | Infiltration | Mitochondria; Chloroplast | Faster and more effective mitochondrial uptake and transfer to the chloroplast | Numata et al., 2020 |
| *N. benthamiana* | KH9-BH100 | DNA, RNA, protein | Infiltration | Leaves | Introduction of many biomolecules simultaneously and effectively | Terada et al., 2020 |
| Lettuce and Rapeseed | Chloroplast and mitochondria targeting peptides fused with cytosine base editor | DNA, RNA | Co-culture | Protoplast | Effective mitochondrial and chloroplast localisation, then targeted and transgene-free base editing | Thagun et al., 2020 |
| *N. tabacum* | Cationic oligopeptide polyarginine  12-mer | RNA | Co-culture | Suspension cells | Efficient post-transcriptional silencing | Kim et al., 2021 |
