# Supplementary material for: Regulation and safety measures for nanotechnology-based agri-products
Source: Front Genome Ed. 2023 Jun 21;5:1200987. doi: 10.3389/fgeed.2023.1200987 (PMC10320728; doi:10.3389/fgeed.2023.1200987)
Supplement: Supplementary file 2 [file Table2.DOCX]

**Table 2.** Application of Nanoparticles in the agriculture.

| **Application** | **Name** | **Composition with** | **Properties/Effect** | **Reference** |
| --- | --- | --- | --- | --- |
| Nanofertilizers | ZnO | Metallic engineered nanoparticles | Grain production improved during crop maturity | Tarafdar et al., 2014 |
|  | CuO | Metallic engineered nanoparticles | Increases nutrient and allicin content in green onion (*Allium fistulosum*). | Wang et al., 2020 |
|  | Fe  Fe3O4 | Metallic engineered nanoparticles | Positive impact on maize (Zea mays) plants growth by increase in photosynthetic rate and chlorophyl content | Jalali et al., 2016 |
|  | CeO_2_ and ZnO | Metallic engineered nanoparticles | Enhances the nutritional value of cucumber (Cucumis sativus) by increase in starch and protein content. | Zhao et al., 2014 |
| Nanopesticiede | Pyrethrins | Temperature-responsive mixed micelle (MMs–Pys–7) | Higher larvicidal effect against *Culex pipiens pallens* at 26 °C | Zhang et al., 2019b |
|  | azoxystrobin | Mesoporous silica nanoparticles-carboxymethyl chitosan (MSN-CMCS) | Better fungicidal effect against *Phytophthora infestans* (tomato late blight) | Xu et al., 2018 |
|  | Novaluron nanoparticles | Nanodispersion | Its insecticidal property against *Spodoptera littoralis* larvae resembles commercial emulsion concentrate formulation | Elek et al., 2010 |
|  | Nanopermethrin | Nanoemulsion | More potent larvicidal property than bulk permethrin | Anjali et al., 2010 |
|  | γ -Cyhalothrin | Solid lipid nanoparticle | Similar insecticidal activity against both *Spodoptera littoralis* larvae and *Dysdercus cingulatus* nymphs but lower Aquatic toxicity than emulsion concentrate. | Frederiksen et al., 2003 |
|  | Imidacloprid | Nanometal + active ingredient | Significantly high toxicity against *Martianus dermestoides* than aqueous formulation. | Guan et al., 2008 |
| Nanotechnology-based plant growth regulators | Nitric acid (NO) | Chitosan nanoparticles | Increase of NO bioactivity under salt stress in maize plants. | Oliveira et al., 2016 |
|  | Gibberellic acid (GA3) | poly(γ-glutamic acid) (γ-PGA) and chitosan (CS) polymers, Nanoparticles | Enhanced seed germination and leaf area in *Phaseolus vulgaris* | Pereira et al., 2017 |
|  | Multi-walled carbon nanotubes (CNTs) | - | Twice flower and fruit production in tomato plant grown in CNTs supplemented soil | Khodakovskaya et al., 2013 |
