# Supplementary material for: Regulation and safety measures for nanotechnology-based agri-products
Source: Front Genome Ed. 2023 Jun 21;5:1200987. doi: 10.3389/fgeed.2023.1200987 (PMC10320728; doi:10.3389/fgeed.2023.1200987)
Supplement: Supplementary file 3 [file Table5.DOCX]

**Table 5.** Some examples of nanomaterials that have been examined and defined by US regulatory bodies on parameters like safety, risk assessment, and efficacy.

| **Nanomaterial** | **Regulatory Body** | **Parameters Studied** | **References** |
| --- | --- | --- | --- |
| Titanium dioxide nanoparticles | US Food and Drug Administration (FDA) | Safety in sunscreens and other topical products | FDA, 2019 |
| Silver nanoparticles | Environmental Protection Agency (EPA) | Safety and risks to human health and the environment | EPA, 2018 |
| Carbon nanotubes | National Institute for Occupational Safety and Health (NIOSH) | Potential health effects and recommended exposure limits | NIOSH, 2013 |
| Quantum dots | EPA | Potential risks to human health and the environment | EPA, 2017 |
| Nanosilver | FDA | Safety and approval for use in medical devices, not yet approved for food or food packaging | FDA, 2019 |
