# Supplementary material for: Regulation and safety measures for nanotechnology-based agri-products
Source: Front Genome Ed. 2023 Jun 21;5:1200987. doi: 10.3389/fgeed.2023.1200987 (PMC10320728; doi:10.3389/fgeed.2023.1200987)
Supplement: Supplementary file 4 [file Table3.DOCX]

**Table 3.** Application of nanosensors in agriculture.

| **Nanosensors** | **Effect** | **References** |
| --- | --- | --- |
| Polyaniline (PANI) and multi-walled carbon nanotubes (MWCNTs) core–shell modified glassy carbon electrode (GC) | Exhibit detection limits upto 0.95 µMol L^-1^ for carbamate pesticides in fruits and vegetables | Cesarino et al., 2012 |
| Rhodamine B-covered gold nanoparticle (RB-AuNP) | Detect various organophosphorus and carbamate pesticides uoto 0.1 μg/L in agriculture products and river water. | Liu et al., 2012 |
| Screen-printed electrode (SPE) modified with a dispersion of carbon black (CB) and chitosan | Detect organophosphorus pesticide; paraoxon upto 0.05 μg L^–1^ in water sample | Talarico et al., 2016 |
| Cadmium telluride quantum dots (CdTe QDs) | Detect organophosphorus pesticides in 10^-12^ to 10^-6^ M | Zheng et al., 2011 |
| Cysteamine modified gold nanoparticles | Detection of nitrates in direct setting of the field | Mura et al., 2015 |
| graphene-based nano-antenna  integrated carbon nano-tubes | Dectection of insect attacks on plants | Afsharinejad et al., 2105 |
