# Supplementary material for: Regulation and safety measures for nanotechnology-based agri-products
Source: Front Genome Ed. 2023 Jun 21;5:1200987. doi: 10.3389/fgeed.2023.1200987 (PMC10320728; doi:10.3389/fgeed.2023.1200987)
Supplement: Supplementary file 5 [file Table4.DOCX]

**Table 4:** The demerits of the Nanotechnology-based agriculture techniques

| **Nanotechnology-based Agriculture Products** | **Demerits** | **References** |
| --- | --- | --- |
| Nanofertilizers | Potential toxicity to plants and soil microorganisms due to accumulation of nanoparticles.  Uncertainty about the long-term environmental effects.  High cost of production. | Wang et al., 2018; Wang et al., 2019; Wang et al., 2020 |
| Nanopesticides | Risk of unintended harm to non-target organisms, including beneficial insects, pollinators, and soil microorganisms.  Potential toxicity to human health.  Concerns about the persistence of nanoparticles in the environment. | Kookana et al., 2011; Kookana et al., 2014; Liu et al., 2016 |
| Nano-based Plant Growth Regulators | Potential toxicity to plants, especially at high concentrations.  Uncertainty about the long-term environmental effects.  Limited knowledge about the mechanism of action. | Barrena et al., 2016; Tripathi et al., 2018; Wang et al., 2020 |
| Nanotechnology in Transgenic Plant Development | Risk of unintended effects on non-target organisms, including insects, wildlife, and soil microorganisms.  Uncertainty about the long-term environmental effects.  Ethical concerns regarding the potential for gene flow to wild populations. | Kuzma and Kokotovich, 2011; Purnell et al., 2018; Huang et al., 2019 |
| CRISPR technology | Potential unintended consequences of gene editing.  Ethical concerns around genetic modification | Pauwels and van der Straeten, 2016; Lander, 2016 |
