# Supplementary material for: Regulation and safety measures for nanotechnology-based agri-products
Source: Front Genome Ed. 2023 Jun 21;5:1200987. doi: 10.3389/fgeed.2023.1200987 (PMC10320728; doi:10.3389/fgeed.2023.1200987)
Supplement: Supplementary file 6 [file Table6.DOCX]

**Table 6.** The regulatory framework on Regulation and Safety Measures for Nanotechnology-based Agri-Products of different countries

| **Country** | **Regulatory Framework** | **Regulatory Body** | **Safety Measures** | **Labelling Requirements** | **References** |
| --- | --- | --- | --- | --- | --- |
| United States | Combination of existing regulations and guidelines from EPA, FDA, and USDA | EPA, FDA, USDA | Safety assessment and management, regulations on labelling of nanomaterials | No mandatory labelling requirements | EPA, 2017; FDA, 2018; USDA, 2016 |
| European Union | Comprehensive regulatory framework, including REACH regulation and regulations governing labelling and pesticide residues | European Commission, European Chemicals Agency, European Food Safety Authority | Safety assessment and management, mandatory labelling requirements | Mandatory labelling of nanomaterials in food and cosmetics | EC, 2018; ECHA, 2021; EFSA, 2018 |
| China | Regulatory framework established by National Nanotechnology Standardization Technical Committee, guidelines for safety assessment and management, labelling, and traceability | Ministry of Agriculture and Rural Affairs | Safety assessment and management, guidelines for labelling and traceability | Guidelines for labelling and traceability | NNSC, 2017; MARA, 2017; SAMR, 2018 |
| India | Limited regulatory framework, voluntary guidelines for safety assessment and management, guidelines for use of nanomaterials in food | Ministry of Environment, Forest and Climate Change, Food Safety and Standards Authority of India | Voluntary guidelines for safety assessment and management, guidelines for use of nanomaterials in food | No mandatory labelling requirements | MoEFCC, 2014; FSSAI, 2018; DST, 2018 |
